# Supplementary material for: Staphylococcus aureus Behavior on Artificial Surfaces Mimicking Bone Environment
Source: Pathogens. 2023 Feb 28;12(3):384. doi: 10.3390/pathogens12030384 (PMC10056644; doi:10.3390/pathogens12030384)
Supplement: Supplementary file 1 [file pathogens-12-00384-s001.zip › pathogens-2198719-supplementary.pdf]

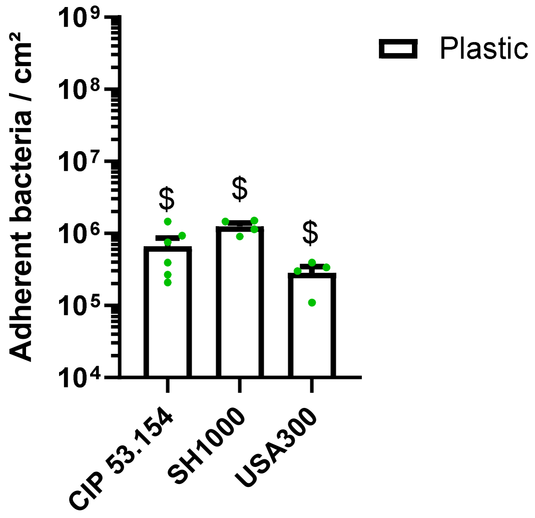

Figure S1. *S. aureus* adhesion on plastic support. Results represent the number of viable adherent bacteria per cm² for CIP 53.154, SH1000 and USA300. *n* = 4 to 8. (green dots represent independent biological replicates). Wilcoxon Man Whitney test; $ = stastically different from collagen and CaP coatings.
